# Emergency management in epidermolysis bullosa: consensus clinical recommendations from the European reference network for rare skin diseases

**DOI:** 10.1186/s13023-020-01403-x

**Published:** 2020-06-06

**Authors:** Jemima E. Mellerio, Maya El Hachem, Nathalia Bellon, Giovanna Zambruno, Hana Buckova, Rudolf Autrata, Carmen Salavastru, Tamara Caldaro, Celine Greco, Cristina Has, Christine Bodemer

**Affiliations:** 1 St John’s Institute of Dermatology, Guy’s and St Thomas’ NHS Foundation Trust, London, UK; 2 Dermatology Unit, Bambino Gesù Children’s Hospital, IRCCS, Rome, Italy; 3 Dermatology Department, reference Centre MAGEC, Necker- Enfants Malades Hospital, Paris-Centre University, Paris, France; 4 Genetics and Rare Diseases Research Division, Bambino Gesù Children’s Hospital, IRCCS, Rome, Italy; 5 Dermatology Department, Children’s Hospital, University Hospital Brno, Brno, Czech Republic; 6 Pediatric Ophthalmology Department, Children’s Hospital, University Hospital Brno, Brno, Czech Republic; 7 Paediatric Dermatology Department, Carol Davila University of Medicine and Pharmacy, Bucharest, Romania; 8 Endoscopy and Digestive Surgery Unit, Bambino Gesù Children’s Hospital, IRCCS, Rome, Italy; 9 Department of Pain and Palliative Medicine, reference Centre MAGEC, Necker- Enfants Malades Hospital, Paris-Centre University, Paris, France; 10 Department of Dermatology, Medical Center, University of Freiburg, Faculty of Medicine, University of Freiburg, Breisgau, Germany; 11 Service de Dermatologie, Hôpital Necker Enfants Malades, 149 rue de Sèvres, 75015 Paris, France

**Keywords:** Epidermolysis bullosa, Blister, Esophageal obstruction, Airway obstruction, Corneal erosion, Urinary retention, Sepsis, Pain, ERN-skin

## Abstract

Epidermolysis bullosa (EB) comprises a group of genetic disorders with the hallmark of fragility of the skin and mucosal surfaces. The severity of different types of EB varies markedly as does the occurrence of extra-cutaneous involvement and complications. A number of emergency situations may occur in the context of EB including obstruction to oral intake from oral or esophageal blisters or scarring, acute airway obstruction, acute urinary retention, sepsis and corneal erosions. Whilst general management principles apply in each of these settings, specific considerations are essential in managing EB to avoid undue trauma or damage to delicate tissues. These recommendations have been developed from a literature review and consensus from experts of the European Network for Rare Skin Disorders (ERN-Skin) to aid decision-making and optimize clinical care by non-EB expert health professionals encountering emergency situations in babies, children and adults with EB.

## Background

Inherited epidermolysis bullosa (EB) comprises clinically and genetically heterogeneous disorders characterized by mechanically-induced mucocutaneous blistering. The main EB types are EB simplex (EBS), junctional EB (JEB), dystrophic EB (DEB) and Kindler EB (KEB). Depending on the level of blister formation, the tissue distribution of the mutated protein and the type of disease-causing mutation, the cutaneous manifestations can be accompanied by mucosal and extracutaneous manifestations and systemic involvement, leading to critical conditions which require rapid decision-making and hospitalization. Such complications and emergency situations can be life-threatening (Table 1). Although every patient with EB may experience such circumstances, those with severe and syndromic subtypes are at higher risk.

**Table 1:** Emergencies in EB

Emergency	Symptoms	EB subtype	Key references
Sepsis	Fever, increased CRP, leukocytosis, positive blood cultures	Any severe EB subtype	[1–3]
Acute feeding inability in newborns/infants	Feeding refusal, sialorrhea, blisters in the oral cavity	DEB	[4, 5]
Acute airway obstruction	Shortness of breath, stridor, distress, dusky skin coloration	JEB	[6–9]
Acute esophageal obstruction	Painful dysphagia, aphagia, sialorrhea, regurgitation	RDEB	[4, 10–12]
Acute urinary retention	Inability to pass urine, lower abdominal distension and pain	JEB, DEB, KEB	[13–16]
Corneal erosion	Pain, photophobia, blepharospasm, tearing	JEB, DEB, KEB	[5, 17–21]

*CRP* C-reactive protein, *DEB* dystrophic epidermolysis bullosa, *RDEB* recessive DEB, *JEB* junctional epidermolysis bullosa, *KEB* Kindler epidermolysis bullosa

In infants with severe EBS, JEB or DEB, widespread blisters and erosions allow bacterial colonization that may lead to sepsis, a common cause of death [1–3]. Oral blisters and erosions are very frequent in all types of EB. Esophageal erosions and strictures are a common feature of DEB, especially in recessive subtypes, but may also occur in KEB. Both oral and esophageal involvement cause nociceptive and neuropathic pain and hamper feeding contributing to failure to thrive and to nutritional deficiencies [4, 22, 23]. Dyspnea due to laryngeal and tracheal lesions with the risk of complete airway obstruction occurs in specific forms of JEB or in EBS with muscular dystrophy [6]. Genitourinary erosions resulting in scarring is common in JEB, DEB and KEB [13, 14]. Finally, ocular mucosal membranes may be affected by painful erosions in JEB, DEB and KEB [17, 18].

A limited number of reference centers for rare diseases exist in each European country. However, emergencies, by definition, occur suddenly requiring prompt management by healthcare professionals who frequently do not belong to a reference center and may not be aware of disease-specific features and related care problems. The objective of these recommendations is to provide the user with information on the best clinical practice in emergency situations which may occur in patients with EB, according to data from the literature and the practical expertise of referral centres for these rare diseases. They should provide support: (i) in decision making for patient management, (ii) for the family and involved physicians, in particular those who do not have EB-specific expertise, and (iii) for long-term surveillance and management of possible complications. Users of these recommendations will be dermatologists, neonatologists, pediatricians, general practitioners, acute physicians, anesthesiologists, gastroenterologists, tracheolaryngologists, urologists and ophthalmologists, nurses, and people living with EB and their families. The consensus recommendation development group consisted of dermatologists and pediatric dermatologists, who are healthcare provider representatives of the European Reference Network-Skin (ERN-skin, https://ern-skin.eu) and the multidisciplinary team members they coordinate.

## Methods

During a meeting of the ERN-Skin held on November 21–232,018 in Rome, Italy, the working group on EB identified the need for the development of recommendations for the management of EB patients in emergency situations which are frequently encountered in these individuals. To identify relevant articles in the literature, a search of NCBI PubMed was performed using the terms ‘epidermolysis bullosa and emergency’ with the search period ending in August 2019. In total, 18 articles were identified, appraised and used for these recommendations. In view of the paucity of available papers, which consisted predominantly of non-evidence-based expert opinion and case reports, the recommendations presented here are largely based on the daily clinical practice in the authors’ expert centers, developed with the assistance of other members of their multidisciplinary teams. Draft recommendations were circulated to the recommendation group for comments and approval then a final version circulated to all ERN-Skin EB working group members for review.

### Basic principles

**Fig. 1:**
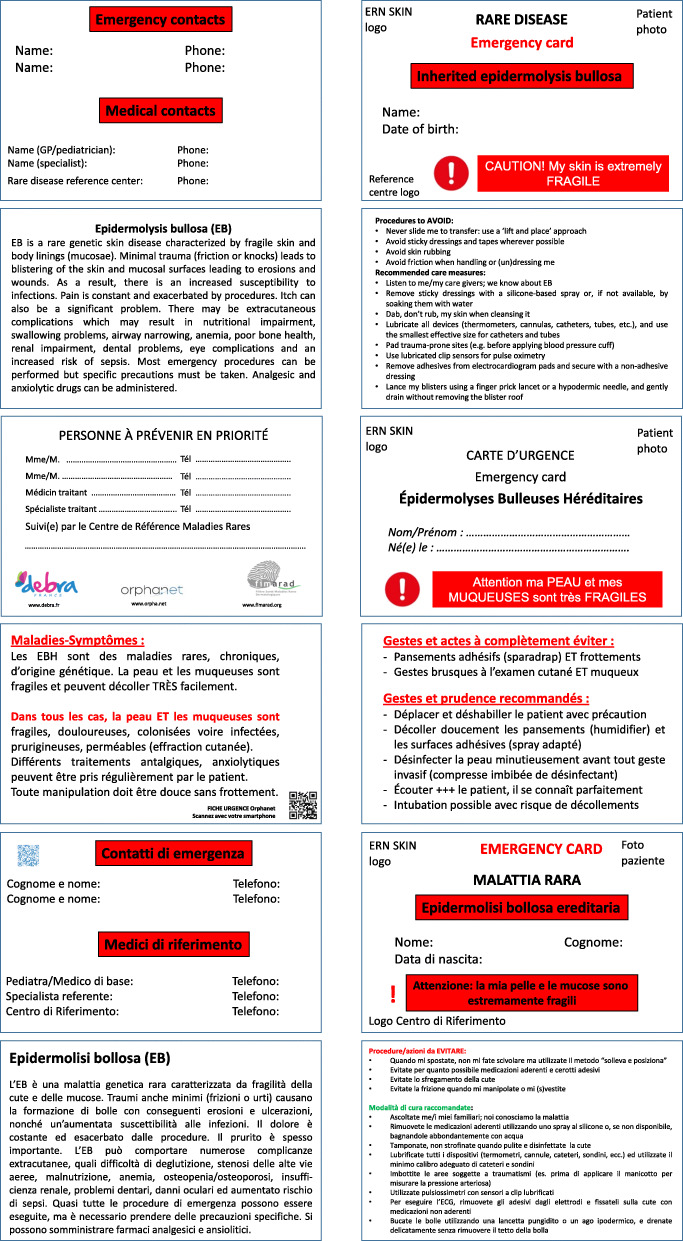
Emergency card for EB patients. In English, French, German and Italian

All individuals with EB have a greater or lesser degree of skin and mucosal fragility. As such, it is imperative that health care professionals dealing with them who are unlikely to be familiar with the condition are made aware of this and the risk of damage, blistering and wounds arising from even gentle handling [5, 24]. Each patient or parents of children with EB should possess a disease-specific emergency card containing basic information on the disease and on EB-specific medical care (Fig. 1).

Where possible, patients should move themselves between surfaces e.g. onto trolleys or beds to avoid the need for lifting which can result in shearing stresses to the skin. The use of rigid slides to laterally transfer patients should be avoided. Babies and small children may be most safely lifted on a pillow or pad to avoid damage.

The use of adhesive tapes e.g. to hold intravenous lines or nasogastric tubes, or sticky electrocardiogram pads should be avoided where possible. If alternatives such as soft-silicone dressings, film or tapes are unavailable, a silicone adhesive removal spray should be used to remove adhesive materials atraumatically from the skin. In neonates, heel prick blood tests should be avoided as shearing stress can cause skin loss when the heel is gripped. Care should be exercised using a tourniquet for venepuncture; a gentle manual grip around the arm with avoidance of shearing stresses to the skin may be used as an alternative.

Blood pressure monitoring may require the use of soft cotton wadding to pad underneath the cuff. Oxygen saturation monitoring should be done with a gentle clip device on the digit or ear as appropriate. An alternative is to use an oxygen probe attached to a cut off plastic glove finger placed onto the patient’s finger if possible.

If a general anesthetic is required in an emergency situation, consideration should be given to the type of intubation and mode of induction [25]. Instruments should be well-lubricated and the patient’s skin protected with dressings to avoid damage from face masks. The eyelids should not be taped closed but should be well-lubricated with eye ointment. A non-adhesive moist dressing may be used on top of this [24].

Mucosal fragility in many forms of EB means that care should be exercised in undertaking invasive procedures such as urinary catheterisation, cystoscopy and laryngobronchoscopy. In most instances, the risks and benefits of procedures must be weighed up and, where possible, advice sought from an EB reference center. Purely exploratory invasive investigations without therapeutic consequence should be avoided. The use of adhesive urine collection bags in babies should be avoided; a clean catch sample (for culture and sensitivity) or cotton wool to collect a sample for dipstick testing is preferred to avoid skin damage.

Whenever the severity of the form of EB justifies primarily palliative care, it is recommended that a palliative care protocol should be drafted in advance by the EB reference center following discussion and approval by the patient (adults) or parents (children), taking into account national regulations. This should be kept at home to be handed over to any emergency response team that is called to a potentially life-threatening emergency before making contact with the reference center that provides care for them.

### Sepsis

#### Definition

Life-threatening organ dysfunction due to a dysregulated host response to infection [26]. In the context of EB, it occurs most commonly in infants with JEB generalized severe where it is a leading cause of death, but less commonly manifests in other forms of EB, particularly recessive DEB (RDEB) generalized severe, JEB generalized intermediate and EBS generalized severe [1–3].

#### Emergency diagnosis

- Clinical history:
  - Malaise and feeling unwell
  - Change in behavior, functioning and level of consciousness
  - Reduced urine output over preceding 12–24 h
  - Risk factors for infection e.g. widespread skin ulceration, indwelling intravenous (IV) lines or urinary catheter, recent interventions or surgery
- Clinical features:

#### NB: clinical scoring systems for different ages should be used to assess an individual’s risk of sepsis

- Unwell, confused, altered conscious level
- Increased respiratory rate (or apnea/grunting in infants)
- Hypotension
- Tachycardia or bradycardia
- Temperature normal, low or raised
- Reduced oxygen saturations on air
- Dusky, mottled skin changes

#### Immediate treatment

- ***Prior to hospitalization:***
- Emergency call
- Oxygen therapy by emergency services if saturations < 90% on air, aiming for saturations of 94–98% (or 88–90% if at risk of hypercapnic respiratory failure)
- Establishment of peripheral IV access by emergency services if possible

- ***At hospital:***
- Use of clinical scoring systems based on vital signs for different ages are invaluable for assessing an individual’s risk of sepsis
- Assessment should be undertaken rapidly so that management can start within one hour from arrival in the emergency department
- Check arterial blood gases including lactate, blood cultures, full blood count, urea and electrolytes, creatinine, coagulation screen, midstream urine and skin swabs for culture and sensitivity, chest radiograph if indicated
- Monitor respiratory rate, heart rate, blood pressure (with an age-appropriate sized cuff), oxygen saturations, temperature and urine output
- Secure peripheral IV access
- Give oxygen if saturations < 90% on air, aiming for saturations of 94–98% (or 88–90% if at risk of hypercapnic respiratory failure)
- Give broad spectrum IV antibiotics once microbiology specimens have been taken
- Give an IV fluid bolus, volume dependent on patient age and cardiac status
- Prepare for transfer to critical care if unstable or signs of deterioration
- Continue to monitor at least every 30 min until clinic situation stabilized

NB: In infants with JEB generalized severe, it is appropriate to offer palliative care in cases of suspected sepsis, based on prior discussions with the family, and dependent on national regulations. To anticipate such situation of emergency, a protocol of palliative care can be written by the specialist of an expert EB centre, following discussion and approval of the parents, and then circulated to the emergency teams.

### Acute feeding inability in newborns/infants

#### Definition

The sudden appearance of large or multiple blisters in the oropharyngeal mucosae or, less commonly, in the esophagus, which prevent feeding. This occurs most frequently in RDEB, but also in EBS generalized severe and JEB [4].

#### Emergency diagnosis

- Sudden uncontrollable crying
- Feeding refusal
- Sialorrhea
- Presence of one or more large tense blisters in the oral cavity

#### Immediate treatment

- ***Prior to hospitalization:***
  - In the case of trained parents/caregivers when there are easily accessible blisters within the oral cavity:
  - Analgesic therapy: paracetamol oral solution 15 mg/kg, which can be repeated up to 4 times/day for the nociceptive component and tramadol hydrochloride 1 mg/kg every 6 h for the neuropathic component)
- Blister lancing using a finger prick lancet or a hypodermic needle or, if not available, a sterilized sewing needle
  - If treatment at home is not recommended (first episode or parents/caregivers not trained) or not effective (failure to drain the blister or immediate relapse, or newborn/infant remaining agitated and crying): management at hospital.
- ***At hospital:***
  - Adequate analgesic therapy (paracetamol oral solution 15 mg/kg 3–4 times a day and tramadol hydrochloride 1 mg/kg every 6 h, and, if not effective, morphine oral solution 0.1–0.3 mg/kg 5–6 times a day [10]. If oral intake is not possible, continuous morphine can be administered at a dose of 10–50 μg/kg/hour via a nasogastric tube (see below)
  - Oral cavity examination and lancing of blister(s) using a finger prick lancet or a hypodermic needle. Prior to lesion lancing, consider midazolam administration (oral or nasal solution0.2–0.5 mg/kg or IV 0.2 mg/kg) in the case of multiple and/or large blisters
  - Immediate follow-up after blister lancing to verify efficacy, and repeat the procedure in case of blisters refilling
- If oral feeding refusal persists, start nasogastric feeding by placement of a small gauge, flexible and soft polyurethane tube lubricated prior to insertion, in order to minimize mucosal trauma [5]
- In case of persistent sialorrhea and feeding difficulty after successful oral lesion treatment, consider the possibility of esophageal involvement (see below)

#### Follow-up

Evaluation by the pediatrician at one week:

- Oral cavity condition
- Nutritional intake and general condition (skin and mucosae color, hydration, weight, length and weight for length)
- Possible associated symptoms such as abdominal pain and signs of gastroesophageal reflux that can worsen oropharyngeal mucosal involvement and needs to be treated medically
- If required, signpost the parents/caregivers to the nearest EB reference or specialized center for ongoing education and follow-up.

### Acute esophageal obstruction

#### Definition

Acute dysphagia and inability to swallow due to the sudden development of obstructive blisters in the hypopharynx/esophagus or to worsening of pre-existing esophageal strictures. This occurs most frequently in RDEB and rarely in KEB and EBS [4, 11].

#### Emergency diagnosis

- Clinical history: frequently onset is during a meal following ingestion of a large or traumatic bolus of food
- Clinical features:
  - Acute complete/almost complete inability to swallow solids or both solids and liquids
  - Acute painful dysphagia
  - Onset of a severe sialorrhea or worsening of a pre-existing sialorrhea
  - Regurgitation

#### Immediate treatment

- ***Prior to hospitalization:***
  - Oral betamethasone 0.1–0.2 mg/kg/day or, in case of (almost) complete swallowing inability, dexamethasone sodium phosphate 0.2% drops 1 mg/kg for up to 2–3 days. If neither are available an equivalent dosage of soluble prednisolone sodium phosphate can be administered. Corticosteroid therapy should be accompanied by administration of oral sodium alginate and sodium bicarbonate solution (from 3 to 12 years 5–10 ml 3–4 times/day; > 12 years 10–15 ml 3–4 times/day)
  - In the case of a complete aphagia, an equivalent dose of corticosteroid should be administrated IV
  - Analgesic therapy if required: paracetamol oral solution (15 mg/kg 3–4 times/day) and tramadol hydrochloride 1 mg/kg every 6 h, and, if not effective, morphine oral solution 0.2–0.3 mg/kg 5–6 times a day, with doses increased by 30% if necessary.
  - Switch to liquid or semi-liquid nutrition, preferably cold, after improvement
  - In case of persistent inability to swallow in infants, lack of improvement within 2–3 days in children and adults, complete dysphagia with inadequate fluid intake or untreatable pain: hospitalization in the nearest EB reference or specialized center.
- ***At hospital:***
  - Adapted analgesic therapy. If necessary, morphine (continuous IV infusion can be used (20 to 50 μg/kg/hour) with regular evaluation and titration of the dose according to response. If pain is not sufficiently relieved, amitriptyline hydrochloride can be used to improve any neuropathic component (continuous IV infusion at an initial dose of 0.3 mg/kg/day)
  - Continue corticosteroid therapy as above
  - Place an IV line for hydration, nutrition and drug administration
  - Esophagogram with water-soluble contrast media (barium should be avoided due to the risk of aspiration into the bronchial tree) should be performed if there is no improvement within a few days. Undertaking this immediately hampers interpretation of results due to acute esophageal edema
  - In case of (sub) total esophageal obstruction detected on an esophagogram, immediate referral to an EB reference center to perform a fluoroscopically-guided balloon esophageal dilatation, followed by oral or IV steroids (as dexamethasone 1 mg/kg twice a day for 3 days) or soluble prednisolone in equivalent dosage) is recommended [7, 12, 27]
  - Evaluation of nutritional status and dietary recommendations [22, 23]
  - Management should be individualized depending on each patient’s presentation and characteristics

#### Follow-up

Within 1, 3 or 6 months, depending on the patient’s previous general condition, evaluation in the EB reference center of:

- nutritional status, weight and growth
- possible associated symptoms, such as abdominal pain and/or signs of gastroesophageal reflux, that could worsen esophageal involvement and require prompt medical treatment
- preventive measures and patient/caregiver education about feeding modalities, food textures and swallowing skills [22, 23].
- Oral viscous budesonide may help reduce the recurrence of esophageal strictures [28, 29]

### Acute upper airway obstruction

#### Definition

Acute upper airway obstruction due to the sudden development of obstructive blisters in the upper airway tract or to worsening of pre-existing trachea-laryngeal granulation tissue/scarring. This is encountered most frequently in JEB generalized severe, JEB with pyloric atresia and JEB laryngo-onycho-cutaneous, and more rarely in EBS with muscular dystrophy and EBS generalized severe [6, 8].

This can lead to acute respiratory failure and always requires emergency hospital admission. This emergency should be anticipated in the most severe forms of EB and, where appropriate and based on national guidelines, a protocol of palliative care, adapted to each patient and situation, written by the expert center multidisciplinary team (including the resuscitation team) with patient/family input and approval should be given to the patient and/or family in advance.

#### Emergency diagnosis

- Clinical history: hoarseness, episodes of respiratory stridor
- Clinical features:
- (Prominent) inspiratory stridor with suprasternal and sternal wall retraction, worsened by crying
- Shortness of breath, agitation and distress
- Pale to dusky complexion

**Immediate intervention:**

- ***Emergency call***

- ***First aid by emergency service (the following procedures are listed according to an escalation therapeutic strategy; the level of intervention depends on the clinical condition of the patient, their response and the EB subtype).***
  1. Proper airway management by positioning via the head tilt-chin lift maneuver
  2. Administer oxygen therapy, secure an IV line and monitor vital signs
  3. Non-invasive ventilation by bag-valve mask
  4. Invasive airway management via intubation (nasal or endotracheal), or emergency tracheostomy
  5. Immediate hospitalization. Whenever possible this should be in the nearest EB reference center but if this would incur delays to treatment, admission to the closest appropriate hospital should be arranged

NB: Depending on local regulations, the procedure(s) to be applied both in the emergency setting and during hospitalization may be available as a written document based on previous discussion and agreement with the parents or patient.

- ***At hospital:***
  - Check the general condition of the patient, the procedures performed by the emergency service, and monitor vital signs
  - Administer oxygen therapy by low or high-flow or continue non-invasive or invasive ventilation (depending on clinical condition)
  - Administer medical therapy (nebulized and/or oral corticosteroids, epinephrine, depending on the clinical condition). Specifically, for mild symptoms: budesonide 2 mg by aerosol which can be repeated, if required, every 20 min up to 3 times, oral dexamethasone 0.6 mg/kg can be added; for moderately severe manifestations: oral dexamethasone 0.6 mg/kg; for severe condition: nebulized epinephrine 0.1 mg/kg, can be repeated every 20 min up to 3 times if required, and oral dexamethasone 0.6 mg/kg, in addition to oxygen therapy [9, 15]
  - Identification of possible triggers (e.g. infection), particularly in children, that may need treatment with antibiotics
  - It may be necessary to reduce the patient’s anxiety with an anxiolytic. A benzodiazepine with a short half-life can be used such as midazolam (50 μg/kg by bolus injection or 250 μg/kg oral administration, repeated as indicated, or amitriptyline hydrochloride oral drops 0.5 mg/kg/day in 3 divided doses, or IV 0.3 mg/kg/day.
  - In case of failure of the above procedures, consider tracheo-laryngeal endoscopy to evaluate upper airway involvement, to remove exuberant granulation tissue/lyse webs and obstructive scars [16, 19]

**NB: palliative care may be considered as an alternative to tracheotomy in infants with JEB generalized severe.**

#### Post-emergency care

- Monitoring of vital signs
- Airway evaluation(s) with flexible nasopharyngoscopy
- Evaluation and medical treatment of possible associated gastroesophageal reflux
- Evaluation of other co-morbidities related to chronicity
- Preventive measures and patient education to early recognize signs of chronic and acute tracheo-laryngeal involvement.

### Acute urinary retention

#### Definition

The inability to voluntarily pass urine leading to acute bladder distention. In EB, this is usually caused by meatal or urethral strictures, or from labial fusion in females. Less commonly, it can result from severe constipation. Acute urinary retention occurs most commonly in JEB, severe RDEB or KEB and can occur at any age from infancy to adulthood [13, 14, 20, 21].

#### Emergency diagnosis

- Clinical history: inability to pass urine, dry diapers in infants, abdominal distension and discomfort There may be a history of difficulty initiating urination and reduced urinary flow, a deflected stream or of blistering around the urethral meatus
- Clinical features:
- Enlarged, tender bladder on abdominal palpation
- Blistering around urethral meatus, meatal stenosis, labial fusion (females)

**Immediate intervention:**

Referral to hospital.

- ***At hospital:***
  - Check urea and electrolytes, creatinine, full blood count and vital signs
  - Analgesia in case of pain: paracetamol oral solution 15 mg/kg/3–4 times a day, and if no improvement, tramadol hydrochloride 1–2 mg/kg every 6 h. Morphine is contraindicated
  - Ultrasound scan of bladder, ureters and kidneys
  - Once acute retention is confirmed on ultrasound, gentle urinary catheterization with a narrow gauge, well-lubricated urinary catheter may be attempted. If unable to pass easily then do not re-attempt
  - If unable to pass a urethral catheter insert a suprapubic catheter
  - Avoid rectal examination to assess constipation or prostatic size to prevent anal blistering

**Post-emergency care:**

- Assess for the cause of the obstruction
- Cystoscopy may be necessary to evaluate urethral strictures and, if possible, should be performed at an EB reference center or with their advice. The procedure should be done only when essential and with the cautious use of a lubricated narrow gauge pediatric cystoscope
- Urethral meatotomy may be performed for meatal strictures
- Surgical labial separation fusion may be carried out for labial fusion
- Dilatation of urethral strictures may be necessary
- Supra-pubic catheters are generally well-tolerated

### Corneal erosions

#### Definition

Erosion or abrasion of the superficial layer of the cornea which is usually extremely painful. This may be acute or chronic and of variable severity. It occurs in all severe EB subtypes, particularly generalized forms of RDEB and all forms of JEB [17, 18].

#### Emergency diagnosis

- Clinical history: pain, inability to open the eye, photophobia and excessive tearing. There may or may not be a history of minor preceding trauma [30].
- Clinical features [17, 18, 30]:
  - Blepharospasm
  - Excessive tearing
  - Redness of the eye
  - Blurred vision
  - Corneal defect(s) visible on fluorescein slit lamp examination

#### Immediate treatment

NB: Care should be taken to avoid shearing damage to the eyelids during ophthalmic examination; the eyes should never be forced open and adhesive tape should be avoided if patching the eyes.

- ***Prior to hospitalization:***
  - Frequent application of preservative-free artificial tear drops, gels or ointments
  - Adequate analgesia (paracetamol oral solution 15 mg/kg/3–4 times a day and tramadol hydrochloride 1 mg/kg every 6 h. If not effective, morphine oral solution 0.2–0.3 mg/kg every 4 h, with doses increased by 30% if necessary.

- ***At hospital:***
  - Topical antibiotic ointments are the first line treatment for healing and prevention of infection (e.g. tobramycin, moxifloxacin or ofloxacin 4–5 times daily) with copious lubricating ointment at night for 7–10 days
  - Preservative-free artificial tear drops or gels every 3–4 h through the day in the long term
  - Cycloplegic eyedrops (e.g. homatropine or scopolamine) may help pain relief
  - Eye patching can be used for prevention of rubbing in infants and small children
  - In the case of large corneal erosions or delayed corneal healing, autologous serum eye drops may be applied, or a bandage contact lens may be used in combination with topical antibiotic
  - If conservative treatment fails, amniotic membrane grafting may be considered

#### Follow-up

Follow up examination every 2 or 3 days until the corneal epithelium is fully healed.

- Long term application of preservative-free artificial tear drops or gels 3–5 times daily and greasy ointment over night
- If corneal scarring or neovascularization is rapidly progressing, topical treatment with corticosteroid eye drops (e.g. fluorometholone) 3–5 times daily may be started but should be rapidly tapered and stopped
- Scleral lens fitting may help to improve visual acuity in cases of corneal irregularities
- In cases of recurrent central erosions, surgical laser phototherapeutic keratectomy (PTK) may be considered, or, with peripheral recurrent erosions, anterior stromal puncture may be considered. These surgical treatments may be associated with overlay amniotic membrane patches due to severe pain and a high risk of persistent epithelial defect
- Bandage contact lenses may also be considered to reduce the risk of recurrent corneal erosions [4, 22]. However, these may require close monitoring and antibiotic prophylaxis [30, 31].

Patients that need care in the operating theater may need protection of the eyes and lids; it is recommended to use lubricating drops or ointment and cover with non-adhesive light moist dressing.

## Conclusion

A number of medical emergencies occur in different forms of EB due to underlying fragility, blistering or scarring of the skin and mucosae, or as a result of other co-morbidities such as extensive skin loss and wounds. Specifically, acute blistering in the mouth or esophagus can cause obstruction to feeding, airway blistering or worsening scarring/granulation tissue can result in potentially life-threatening respiratory obstruction, and blisters or strictures of the genitourinary tract can cause acute urinary retention. Corneal erosions present with acute onset of marked eye pain and need prompt treatment to alleviate symptoms and minimize longer term sequelae. Sepsis occurs more commonly in EB especially when individuals have potential sources of infection such as widespread wounds, indwelling lines or urinary complications. Although the acute management of these emergencies should follow the same basic principles as in non-EB situations, specific care should be taken to avoid undue damage to the skin and mucosae through the interventions performed. The urgent nature of these complications means that it is often not possible to provide the required care in an EB reference center but the recommendations presented here should assist the non-specialist to deal safely and appropriately with emergency situations in EB until advice and/or treatment can be sought from the patient’s EB care team.

## Data Availability

Not applicable.

## Abbreviations

DEB: Dystrophic epidermolysis bullosa
EB: Epidermolysis bullosa
EBS: Epidermolysis bullosa simplex
ERN: European Reference Network
IV: Intravenous
JEB: Junctional epidermolysis bullosa
KEB: Kinder epidermolysis bullosa
PTK: Phototherapeutic keratectomy
RDEB: Recessive dystrophic epidermolysis bullosa
